# Plasma adiponectin levels are associated with circulating inflammatory cytokines in autoantibody positive first-degree relatives of rheumatoid arthritis patients

**DOI:** 10.1371/journal.pone.0199578

**Published:** 2018-06-25

**Authors:** Jan M. Hughes-Austin, Kevin D. Deane, Jon T. Giles, Lezlie A. Derber, Gary O. Zerbe, Dana M. Dabelea, Jeremy Sokolove, William H. Robinson, V. Michael Holers, Jill M. Norris

**Affiliations:** 1 Department of Orthopaedic Surgery, School of Medicine, University of California, San Diego, La Jolla, California, United States of America; 2 Department of Rheumatology, School of Medicine, University of Colorado Anschutz Medical Campus, Aurora, Colorado, United States of America; 3 Division of Rheumatology, College of Physicians and Surgeons, Columbia University, New York, New York, United States of America; 4 Department of Biostatistics and Informatics, Colorado School of Public Health, University of Colorado Anschutz Medical Campus, Aurora, Colorado, United States of America; 5 Department of Epidemiology, Colorado School of Public Health, University of Colorado Anschutz Medical Campus, Aurora, Colorado, United States of America; 6 VA Palo Alto Health Care System, Palo Alto, California and the Division of Immunology and Rheumatology, Stanford University School of Medicine, Stanford, California, United States of America; SERGAS and IDIS, SPAIN

## Abstract

**Background:**

Extra-articular manifestations of rheumatoid arthritis (RA), potentially due to systemic inflammation, include cardiovascular disease and sarcopenic obesity. Adiponectin, an adipose-derived cytokine, has been implicated in inflammatory processes in RA, but little is known regarding its association with inflammation in a pre-clinical period. Therefore, we investigated whether adiponectin was associated with inflammatory markers in individuals at risk for RA, and whether RA-related autoimmunity modifies these associations.

**Methods:**

We analyzed samples from 144 first-degree relatives (FDRs) of RA probands, of whom 23 were positive for anti-cyclic citrullinated peptide antibody and/or ≥ 2 rheumatoid factor isotypes (IgM, IgG or IgA). We called this phenotype the ‘high risk autoantibody profile (HRP)’ as it has been shown in prior work to be >96% specific for future RA. We measured adiponectin, cytokines, and high-sensitivity C-reactive protein (hsCRP). Using linear mixed effects models, we evaluated interaction between HRP positivity and adiponectin on inflammatory markers, adjusting for age, sex, ethnicity, body mass index, pack-years smoking, and use of cholesterol-lowering medications.

**Results:**

In everyone, adiponectin concentration was inversely associated with hsCRP and IL-1β in adjusted models, where a 1% higher adiponectin was associated with a 26% lower hsCRP (p = 0.04) and a 26% lower IL-1β (p = 0.04). Significant interactions between HRP and adiponectin for associations with GM-CSF, IL-6, and IL-9 were detected in fully adjusted models (p = 0.0006, p = 0.006, p = 0.01, respectively). In HRP positive FDRs but not HRP negative FDRs, a 1% higher adiponectin was associated with 97% higher GM-CSF, 73% higher IL-6, and 54% higher IL-9 concentrations.

**Conclusions:**

Adiponectin associates with inflammatory markers, and these associations differ in individuals with a high-risk autoantibody profile compared with those without. The interaction between adiponectin and autoimmunity warrants further investigation into the potential systemic effects of RA-related autoantibodies and adiponectin on inflammation in the absence of clinically apparent RA.

## Introduction

Rheumatoid arthritis (RA) is a chronic systemic autoimmune disease characterized in a majority of cases by the presence of circulating autoantibodies and abnormalities of markers of inflammation. While the primary manifestation of RA is synovial joint swelling, pain, and deformity, extra-articular manifestations of RA include cardiovascular disease and sarcopenic obesity, which are thought to be related to the systemic inflammation in RA. Multiple studies have shown that RA-autoantibodies, as well as cytokines and chemokines, appear up to 14 years before the onset of RA, suggesting a clear ‘pre-clinical’ period of RA.[1–4] Given this pre-clinical period of autoimmunity and inflammation, and altered body composition where muscle mass is lost and adipose tissue is preserved, regardless of changes in body weight, we hypothesized that adiponectin, a cytokine secreted by adipose tissue, may play a role in this pre-clinical inflammatory period.

In individuals without autoimmune diseases, adiponectin has been shown to possess anti-inflammatory, anti-atherogenic, and anti-diabetic properties, as it reduces circulating fatty acid concentrations and triglyceride levels in muscle and the liver.[5] Adiponectin shares strong homologies with TNF-α and complement factor C1q, and has been shown to prevent the transformation of macrophages into foam cells and down-regulate TNF-dependent expression of several adhesion molecules,[6] thus dampening atherogenic processes. In contrast, however, higher concentrations of adiponectin have been observed in patients with chronic autoimmune diseases such as RA and systemic lupus erythematosus (SLE), and have been directly associated with joint damage in RA.[5, 7, 8] Thus, adiponectin plays a complicated role in modulating the innate immune system and inflammation—in some states being anti-inflammatory, but in others perhaps contributing to inflammation and tissue destruction, and has prompted this investigation.[5, 8]

Previous studies have suggested an active role for adiponectin in inflammatory, matrix-destructive, and fibrotic processes contributing to joint destruction in RA[5]. Little is known, however, about adiponectin in the pre-clinical period of RA, and specifically whether autoimmunity modifies associations between adiponectin and inflammatory markers. Therefore, in an effort to identify novel pathways that could be targeted for intervention in the pre-clinical period of RA in order to avert clinical disease, we investigated whether adiponectin was associated with inflammatory markers in individuals at risk for RA, and whether autoimmunity modifies these associations.

## Materials and methods

Studies of the Etiology of Rheumatoid Arthritis (SERA) is a multi-center prospective study in the United States [with sites in New York, Chicago, Omaha (the center of the Rheumatoid Arthritis Investigational Network), Denver, Seattle, and Los Angeles] that is following first-degree relatives (FDRs) of probands with RA. SERA was designed to examine the role of environmental and genetic factors in the development and progression of RA-related autoimmunity, and to explore pre-clinical immunological changes and pathophysiological processes in the absence of confounders such as treatments or secondary complications of active disease. [9, 10] FDRs [parent, sibling, or offspring] of RA patients were recruited through their probands [identified from academic centers, Veterans’ hospitals, and rheumatology clinics] or through responses to advertising. FDRs were eligible to participate if they did not have an RA diagnosis, defined by the 1987 ACR criteria and the 2010 EULAR/ACR criteria, and were ≥18 years old. Institutional Review Boards at all SERA sites approved this study [Colorado Multiple Institutional Review Board, University of Colorado Anschutz Medical Campus, Aurora, CO, USA; Benaroya Research Institute Institutional Review Board, Seattle, WA, USA;Cedars-Sinai Medical Center Institutional Review Board, Los Angeles, CA, USA; Rheumatoid Arthritis Investigational Network [RAIN] at the University of Nebraska Medical Center Institutional Review Board, Omaha, NE, USA; The Feinstein Institute for Medical Research, Human Research Protection Program Institutional Review Board, Manhasset, NY, USA; University of Chicago Institutional Review Board, Chicago, IL, USA], and all participants provided written informed consent.

At research visits, FDRs completed disease and exposure assessment questionnaires, including smoking status and medication use, as well as anthropometric measurements (e.g., height and weight), underwent a standardized interview and 68-count joint examination by a trained clinician, and had blood drawn. All serum samples were tested for anti-cyclic citrullinated peptide (anti-CCP2) antibodies, rheumatoid factor (RF) by nephelometry, and RF isotypes immunoglobulin (Ig) A, G, and M (RF-IgA, RF-IgG, RF-IgM), as described previously.[9] For each visit, we determined positivity for a high-risk autoantibody profile (HRP), which is defined as testing positive for anti-CCP2 and/or two RF isotypes, and has been shown to be 96% specific for future RA.[11]

For this ancillary study, we analyzed 144 FDRs who had complete measures of RA-related autoantibodies, adiponectin, seven cytokines that have been shown to be associated with RA-related autoantibodies or adiponectin,[5, 10] and covariates in samples that had been immediately processed, as delayed sample processing may alter cytokine levels. Of these 144 FDRs, 23 were positive for HRP (HRP(+)), and 121 were negative (HRP(-)). Over a span of six years of follow up, there were a total of 35 HRP(+) samples and 222 HRP(-) samples, with approximately 2 ± 1 study visits per FDR.

Total adiponectin (ng/mL) was measured in plasma using the Millipore Milliplex Human Adiponectin Radioimmunoassay (Millipore, Billerica, Massachusetts, USA). Serum hsCRP was tested by nephelometric assay (BN II Nephelometer, Dade Behring, Deerfield, Illinois, USA); and serum cytokines were tested using a bead-based multiplex assay (Beadlyte kit, Upstate, Charlottesville, Virginia, USA), the Luminex xMAP 100IS platform (Luminex, Austin, Texas, USA), and BioPlex array reader (Bio-rad Laboratories, Hercules, California) with Luminex fluorescent bead technology, using Heteroblock^TM^ (Omega Biologicals Inc., Bozeman, Montana)[12], as described previously.[10, 13] The data discussed in this publication have been deposited in NCBI’s Gene Expression Omnibus (Edgar et al., 2002) and are accessible through GEO Series accessesion number GSE114043 (https://www.ncbi.nlm.nih.gov/geo/query/acc.cgi?acc=GSE114043).

We first evaluated demographic differences by HRP positivity using analysis of covariance with repeated measures for continuous variables and chi-square tests for proportions. Second, we examined whether adiponectin and inflammatory marker concentrations differed by HRP positivity using analysis of covariance with repeated measures. Third, we tested associations between adiponectin and selected inflammatory markers, and further tested for interactions between HRP positivity and adiponectin concentration by including an interaction term when examining the outcome of inflammation. In our analyses with repeated measures, we used linear mixed-effects models [SAS (version 9.2) PROC MIXED] to account for multiple records per subject collected over time while adjusting for age, sex, ethnicity, body mass index (BMI) calculated as weight (kg)/height (m^2^), smoking pack-years, and current cholesterol-lowering medication use, as these medications have been shown to be associated with inflammatory markers. Mixed models provide the best available method to adjust for differing numbers of visits per subject by estimating the variability between subjects and the variability between repeated measurements on the same subject separately, and then using functions of these variance estimates as weights to determine the best estimate of association. In our analysis, both between subject effects and within subject effects (residuals) were normally distributed. We selected 7 individual cytokines that had been previously associated with HRP [IL-2, IL-6, IL-9, GM-CSF, and IFN- γ[10], those shown to be associated with adiponectin [TNF-α, IL-6, IL-1β and IFN- γ[5], and another well-recognized marker of inflammation [hsCRP]. No adjustments were made for multiple comparisons, as our *a priori* hypothesis focused on these specific inflammatory markers.[14] Adiponectin and the inflammatory markers were natural log-transformed to satisfy assumptions of distribution normality. Inflammatory markers were standardized, such that interpretations pertain to a standard deviation (SD) difference of the marker. These regression coefficients can be interpreted as % change in the inflammatory marker per 1% increase in adiponectin.

## Results

The 144 FDRs evaluated in this analysis were predominantly female (74%), non-Hispanic white (82%), and never-smokers (65%). At baseline, FDRs were 49 ± 18 years old, BMI was 28 ± 6, and 11% used cholesterol-lowering medications. There were no significant demographic differences by HRP status. Differences in these descriptive characteristics by HRP phenotype are presented in Table 1.

**Table 1:** Population characteristics by high-risk autoantibody profile (HRP) phenotype in 257 serum/plasma samples from clinic visits of 144 FDRs from the studies of the Etiology of rheumatoid arthritis (SERA) cohort.

	HRP(+) FDRs† (n = 35 visits by 23 FDRs)	HRP(-) FDRs† (n = 222 visits by 121 FDRs)	P-value**
Age, years	47 (16)	49 (17)	0.68
BMI, kg/m^2^	26 (5)	28 (6)	0.56
Gender, % female	80	72	0.33
Non-Hispanic White, %	86	81	0.51
Current use of cholesterol-lowering medications, %	11	14	0.64
Ever smoking cigarettes, %	29	35	0.48

**†**High Risk Autoantibody Profile (HRP) is defined as positivity for anti-CCP2 and/or two or more RF isotypes, and has been shown in prior work using pre-clinical RA samples to be >96% specific for future RA.

**Differences between HRP(+) versus HRP(-) clinic visits were tested using analysis of covariance with repeated measures.

For descriptive purposes, we present median (25^th^, 75^th^ percentile) levels of adiponectin and selected inflammatory markers by whether the FDR serum samples for each clinic visit were HRP(+) versus HRP(-). Adiponectin, hsCRP, IL-1β, IFN-γ, and TNF-α concentrations did not differ significantly by HRP positivity (Table 2). Concentrations of IL-2, IL6, IL-9, and GM-CSF, however, were higher in HRP(+) FDRs compared to HRP(-) FDRs (Table 2).

**Table 2:** Median (25th, 75th Percentile)* levels of adiponectin and inflammatory markers by high-risk autoantibody profile (HRP) phenotype in 257 serum/plasma samples from clinic visits of 144 FDRs from the studies of the Etiology of rheumatoid arthritis (SERA) cohort.

	HRP(+) FDRs† (n = 35 visits by 23 FDRs)	HRP(-) FDRs† (n = 222 visits by 121 FDRs)	P-value**
**Adiponectin**	12.4 (8.8, 15.7)	11.6 (7.2, 16.1)	0.72
**hsCRP**	1.2 (0.5, 4.0)	1.8 (0.6, 4.3)	0.3141
**IL-1**β	1.6 (1.2, 2.0)	1.3 (1.0, 1.7)	0.7517
**IL-2**	7.7 (0, 20.5)	0 (0, 5.7)	0.002
**IL-6**	7.1 (5.1, 11.7)	5.3 (4.0, 7.3)	0.0043
**IL-9**	59.5 (22.3, 894.6)	21.6 (14.4, 58.0)	<.0001
**GM-CSF**	31.8 (20.2, 72.8)	19.3 (12.4, 37.8)	0.0257
**IFN-**γ	61.3 (38.3, 99.6)	40.9 (28.3, 64.2)	0.133
**TNF-**α	5.6 (0, 22.7)	4.9 (0, 14.1)	0.2487

*These median and 25^th^ and 75^th^ percentile estimates are raw values.

**†**High Risk Autoantibody Profile (HRP) is defined as positivity for anti-CCP2 and/or two or more RF isotypes, and has been shown in prior work using pre-clinical RA samples to be >96% specific for future RA.

**Differences between HRP(+) versus HRP(-) clinic visits were tested using analysis of covariance with repeated measures. Subsequent analysis utilizes a natural log-transformed and standardized value for each of these markers.

In all subjects, hsCRP and IL-1β were significantly inversely associated with adiponectin concentration in adjusted models, where a 1% higher adiponectin was associated, on average, with a 26% lower standardized hsCRP (p = 0.04) and a 26% lower IL-1β (p = 0.04). IL-2, IFN-γ, and TNF-α were inversely associated with adiponectin concentration in adjusted models as well, but these associations were not statistically significant. (Table 3).

**Table 3:** Association between adiponectin and markers of inflammation in 257 serum/plasma samples from clinic visits of 144 FDRs from the studies of the Etiology of rheumatoid arthritis (SERA) cohort.

Markers of Inflammation	β (SD)*	p-value
**hsCRP**	-0.2617 (0.13)	0.0431
**IL-1**β	-0.2644 (0.13)	0.0434
**IL-2**	-0.1040 (0.14)	0.4539
**IFN-**γ	-0.1395 (0.14)	0.3062
**TNF-**α	-0.2179 (0.13)	0.1053

*Adjusted for HRP status, age, sex, ethnicity, BMI, pack-years of smoking, and current use of cholesterol-lowering medications.

Regression coefficients can be interpreted as % difference in the inflammatory marker per 1% higher adiponectin, e.g., a 1% higher adiponectin resulted in a 26% lower standardized hsCRP.

**Fig 1:**
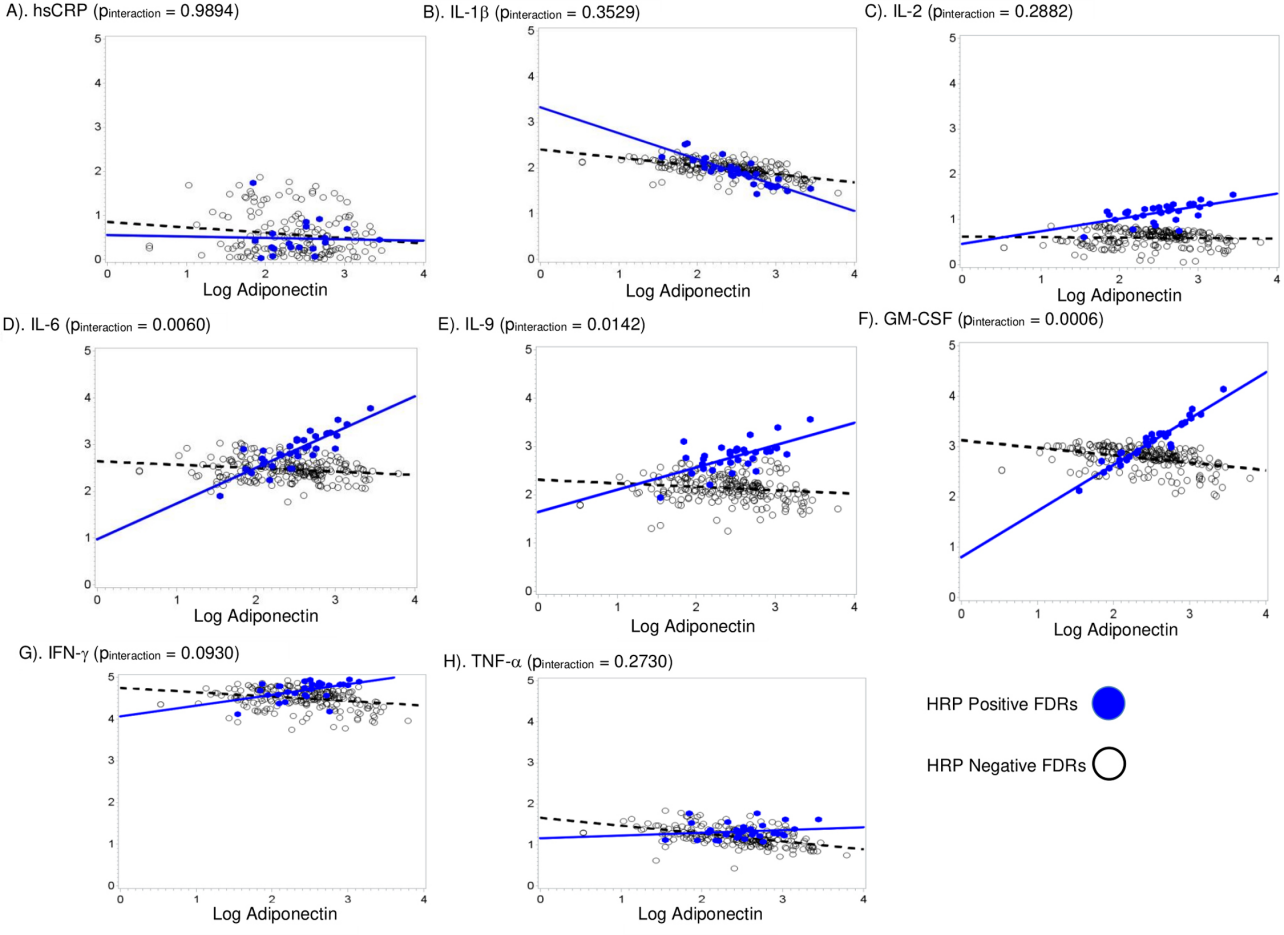
Modification of the association between adiponectin and inflammatory markers (A through H) by HRP status using linear mixed models in the studies of the Etiology of rheumatoid arthritis. This figure presents the interaction between adiponectin and High-Risk profile autoantibody (HRP) status in 257 serum and plasma samples from clinic visits of 144 first degree-relatives of RA patients in the Studies of the Etiology of Rheumatoid Arthritis. All analyses were adjusted for age, sex, ethnicity, BMI, pack-years of smoking, and current use of cholesterol-lowering medications.

Significant interactions between HRP and adiponectin for associations with GM-CSF, IL-6, and IL-9 were detected in fully adjusted models (p = 0.0006, p = 0.006, p = 0.01, respectively), where slopes were estimated for associations between adiponectin and inflammatory markers for HRP(+) and HRP(-) FDRs. These interactions suggest that in HRP(+) FDRs, higher concentrations of adiponectin were associated with higher levels of GM-CSF, IL-6, and IL-9, while no associations were observed in HRP(-) FDRs. (Fig 1). Specifically, in HRP positive FDRs but not HRP negative FDRs, a 1% higher adiponectin was associated with 97% higher GM-CSF, 73% higher IL-6, and 54% higher IL-9 concentrations. (Fig 1)

## Discussion

In FDRs who were positive for a high-risk autoantibody profile specific for future RA, higher concentrations of adiponectin were significantly associated with higher concentrations of three pro-inflammatory cytokines, GM-CSF, IL-6, and IL-9. These associations were absent, however, in FDRs who did not have the high-risk autoantibody profile. Independent of the HRP status, inflammatory markers hsCRP and IL-1β were inversely associated with adiponectin, which is consistent with the putative anti-inflammatory characteristics of adiponectin. Taken together, these findings encourage further investigation into associations between adiponectin and inflammatory markers in various environments of autoimmunity.

Several investigators have reported adiponectin’s direct relationship with pro-inflammatory cytokines in chronic inflammatory diseases such as RA and SLE [5, 15], implying that adiponectin exerts pro-inflammatory effects on effector cells. In particular, in vitro studies have shown that when stimulated with adiponectin, RA synovial fibroblasts secreted increased concentrations of pro-inflammatory cytokines, such as IL-6, as well as various chemokines.[5] Our findings extend these reports by suggesting that autoimmunity, even in the absence of clinical RA, modifies adiponectin’s associations with inflammatory markers and potentially its actions on effector cells. Further, our observation that the high-risk autoantibody profile modified the association between adiponectin, IL-6, IL-9, and GM-CSF, three markers of systemic inflammation, and not other inflammatory markers, i.e., hsCRP, IL-1β, IL-2, INF-γ, and TNF-α, which are indicative of acute inflammation, suggests that the interaction between adiponectin and the high-risk profile may occur systemically. We did not investigate this interaction between adiponectin and the high-risk autoantibody profile at the level of the joints, and thus cannot draw conclusions regarding local/acute inflammation based on the current results. Our results, however, lead us to further pursue future research directions regarding the role of adiponectin in local versus systemic inflammation in the setting of autoimmune disease.

Adiponectin concentration is closely associated with visceral adiposity, which we did not measure. In addition, we did not measure lean body mass, and were therefore not able to account for body composition in our analysis. Instead, we adjusted all analyses for BMI, which may have been an under adjustment for adiposity. It is unlikely that our failure to measure visceral adiposity resulted in an overestimation of adiponectin’s association with inflammatory markers, as multiple cell types [e.g. white and brown adipocytes, mucosal cells, liver, skeletal muscle cells, cardiomyocytes, and salivary gland epithelial cells[16]] produce adiponectin, and visceral adipose tissue does not account for all circulating adiponectin.

In this study, we did not use a population control group due to the inaccessibility of appropriate samples. Instead we utilized an internal control (i.e., autoantibody negative (HRP(-)) FDRs from the cohort), which has many methodological advantages, including the comparability of data acquisition and blood processing, and the lack of selection biases inherent in external controls. Following FDRs without classifiable RA also allowed us to examine associations between adiponectin, autoimmunity, and inflammatory markers in a population that is not receiving treatment for RA, which prevents potential confounding by indication. Another strength was that we analyzed cytokines as continuous variables. This analysis allowed us to observe potentially more subtle relationships between these inflammatory markers and adiponectin, which may be more appropriate in individuals without clinically apparent articular disease. We recognize that our results are not generalizable to individuals of normal BMI, 18.5–24.9 kg/m^2^, and to autoantibody-positive individuals who are not first-degree relatives of RA patients as this particular study investigated only first-degree relatives with an average BMI of 28 ± 6 kg/m^2^. Future investigations of various BMI categories as well as associations between adiponectin and cytokines and modification by autoantibody status in non-first-degree relatives would address whether the findings in the present study persist in other populations.

Our adiponectin assay did not distinguish between high- (HMW), middle- (MMW), or low- (LMW) molecular weight adiponectin. Previous research has demonstrated that LMW adiponectin displays anti-inflammatory properties whereas HMW may display more pro-inflammatory effects, and is thought to be a more active form of adiponectin than total adiponectin in the context of metabolic diseases[7]. Therefore, our results may underestimate potential pro-inflammatory relationships between adiponectin and markers of inflammation, which deserves to be explored in future studies.

## Conclusions

In conclusion, in a population without RA, but at increased risk for future RA, adiponectin associates differentially with inflammatory markers according to autoimmunity status, evidenced by its positive associations with pro-inflammatory cytokines solely in HRP positive FDRs. These findings, in combination with prior studies, suggest that an environment of autoimmunity may alter the role of adiponectin, and warrants further investigation into the potential systemic effects of RA-related autoantibodies and adiponectin on inflammation in the absence of clinically apparent RA.
